# Outbreak of Ebola Virus Disease in Guinea: Where Ecology Meets Economy

**DOI:** 10.1371/journal.pntd.0003056

**Published:** 2014-07-31

**Authors:** Daniel G. Bausch, Lara Schwarz

**Affiliations:** 1 Tulane School of Public Health and Tropical Medicine, New Orleans, Louisiana, United States of America; 2 United States Naval Medical Research Unit No. 6, Lima, Peru; 3 McGill University, Montreal, Canada

**Figure 1:**
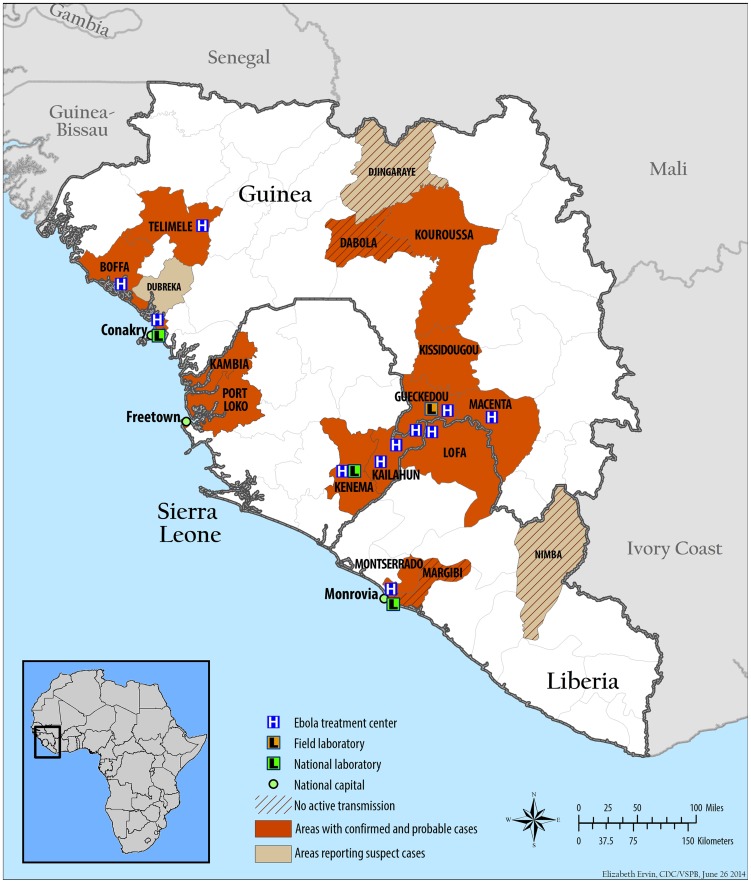
Map of the three countries (Guinea, Liberia, and Sierra Leone) involved in the 2013–2014 outbreak of Ebola virus disease as of June 20, 2014. The putative first virus introduction and epicenter are in the vicinity of the town of Guéckédou in the Guinea Forest Region. CDC: http://www.cdc.gov/vhf/ebola/resources/distribution-map-guinea-outbreak.html.

Ebola virus is back, this time in West Africa, with over 350 cases and a 69% case fatality ratio at the time of this writing [1]. The culprit is the Zaire ebolavirus species, the most lethal Ebola virus known, with case fatality ratios up to 90%. The epicenter and site of first introduction is the region of Guéckédou in Guinea's remote southeastern forest region, spilling over into various other regions of Guinea as well as to neighboring Liberia and Sierra Leone (Figure 1). News of this outbreak engenders three basic questions: (1) What in the world is Zaire ebolavirus doing in West Africa, far from its usual haunts in Central Africa? (2) Why Guinea, where no Ebola virus has ever been seen before? (3) Why now? We'll have to wait for the outbreak to conclude and more data analysis to occur to answer these questions in detail, and even then we may never know, but some educated speculation may be illustrative.

**Figure 2:**
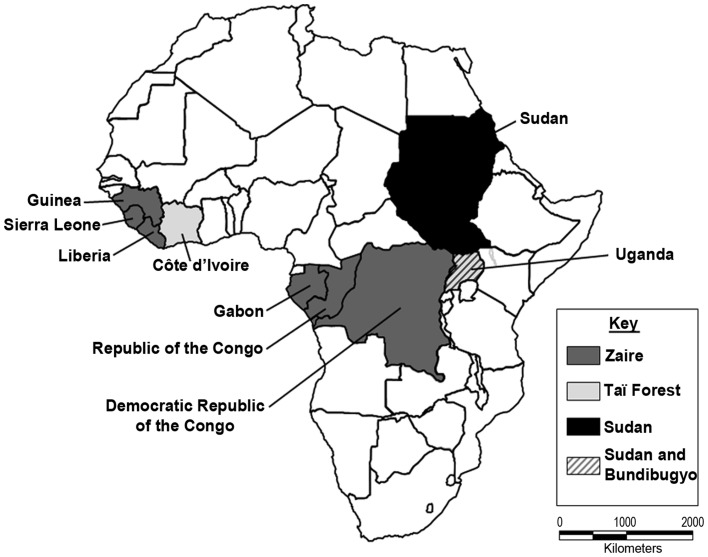
African countries where endemic transmission of Ebola virus has been noted.

The *Ebolavirus* genus is comprised of five species, Zaire, Sudan, Taï Forest, Bundibugyo, and Reston, each associated with a consistent case fatality and more or less well-identified endemic area (Figure 2). Zaire ebolavirus had been previously found only in three Central African countries—the Democratic Republic of the Congo, Republic of the Congo, and Gabon. Thus, the logical assumption when Ebola virus turned up in Guinea was that this would be the Taï Forest species previously noted in Guinea's neighbor, Côte d'Ivoire.

How did Zaire ebolavirus get all the way over to West Africa? The two possibilities appear to be that the virus has always been present the region, but we just never noticed, or that it was recently introduced. The initial report and phylogenetic analyses on the Guinea outbreak suggested that the Zaire ebolavirus found in Guinea is a distinct strain from that noted in Central Africa [1], thus suggesting that the virus may not be a newcomer to the region. However, subsequent reworking and interpretations of the limited genetic data have cast some doubt on this conclusion [2]. If Zaire ebolavirus had been circulating for some time in Guinea, one might expect greater sequence variation than the 97% homogeneity noted relative to that isolated from Central Africa [1].

Phylogenetic arguments aside, if Ebola virus was present in Guinea, wouldn't we have seen cases before? Not necessarily. Many pathogens may be maintained in animals with which humans normally have little contact, thus providing limited opportunity for infection. Furthermore, the proportion of infected animals may often be very low, so even frequent contact may not result in pathogen transmission. Even if human Ebola virus infection has occurred, it may not be recognized; contrary to popular concept, the clinical presentation of viral hemorrhagic fever is often very nonspecific, with frank bleeding seen in a minority of cases, so cases may be mistaken for other, more common diseases or, in the case of Guinea, Lassa fever, which is endemic in the area of the outbreak [3]. Nor are laboratory diagnostics routinely available in West Africa for most viral hemorrhagic fevers [4]. Ebola virus testing of human serum samples collected as far back as 1996 as part of surveillance for Lassa fever in the same region as the current outbreak could help reveal whether humans had exposure to Ebola virus prior to this outbreak [3]. We are presently organizing with collaborators to conduct ELISA antigen testing, PCR, and cell culture for Ebola virus on samples from persons who met the case definition for viral hemorrhagic fever but tested negative for Lassa fever. We will also test all samples for IgG antibody to Ebola virus to explore the prevalence of past exposure.

Could Zaire ebolavirus have been recently introduced into Guinea from Central Africa? Introduction from a human traveler seems unlikely; there is little regular travel or trade between Central Africa and Guinea, and Guéckédou, the remote epicenter and presumed area of first introduction, is far off the beaten path, a minimum 12 hour drive over rough roads from the capitals of Guinea, Liberia, or Sierra Leone (Figure 1). Furthermore, with the average incubation period as well as time from disease onset until death in fatal cases both a little over a week, a human traveler would have to make the trip from Central Africa to Guéckédou rather rapidly.

If Ebola virus was introduced into Guinea from afar, the more likely traveler was a bat. Although a virus has not yet been isolated, PCR and serologic evidence accumulated over the past decade suggests that fruit bats are the likely reservoir for Ebola virus. The hammer-headed fruit bat (*Hypsignathus monstrosus*), Franquet's epauletted fruit bat (*Epomops franqueti*), and the little collared fruit bat (*Myonycteris torquata*) are among the leading candidates [5]–[9]. Many of these species are common across sub-Saharan Africa, including in Guinea, and/or may migrate long distances, raising the possibility that one of these wayward flyers may have carried Ebola virus to Guinea [8]. Introduction into humans may have then occurred through exposures related to hunting and consumption of fruit bats, as has been suspected in Ebola virus outbreaks in Gabon [8]. Similar customs have been reported in Guinea, prompting the Guinean government to impose a ban on bat sale and consumption early on in the outbreak. Field collections and laboratory testing for Ebola viruses of bats collected from the Guinea forest region should shed light on the presence or absence of these various species in the area and possible Ebola virus infection. Indeed, a team of ecologists is already on the ground beginning this work.

But why Guinea and why Guéckédou? Certainly this is not the only place bats migrate. Unfortunately, Ebola virus outbreaks typically constitute yet another health and economic burden to Africa's most disadvantaged populations. Despite the frequently promulgated image of Ebola virus mysteriously and randomly emerging from the forest, the sites of attack are far from random; large hemorrhagic fever virus outbreaks almost invariable occur in areas in which the economy and public health system have been decimated from years of civil conflict or failed development [10]–[13]. Biological and ecological factors may drive emergence of the virus from the forest, but clearly the sociopolitical landscape dictates where it goes from there—an isolated case or two or a large and sustained outbreak.

The effect of a stalled economy and government is 3-fold. First, poverty drives people to expand their range of activities to stay alive, plunging deeper into the forest to expand the geographic as well as species range of hunted game and to find wood to make charcoal and deeper into mines to extract minerals, enhancing their risk of exposure to Ebola virus and other zoonotic pathogens in these remote corners. Then, the situation is compounded when the unlucky infected person presents to an impoverished and neglected healthcare facility where a supply of gloves, clean needles, and disinfectants is not a given, leaving patients and healthcare workers alike vulnerable to nosocomial transmission. The cycle is further amplified as persons infected in the hospital return to their homes incubating Ebola virus. This classic pattern was noted in Guinea, where early infection of a healthcare worker in Guéckédou triggered spread to surrounding prefectures and eventually to the capital, Conakry [1]. Lastly, with an outbreak now coming into full force, inefficient and poorly resourced governments struggle to respond, as we are seeing all too clearly with this outbreak of Ebola virus disease in West Africa, which is now by far the largest on record. The response challenge is compounded in this case by infected persons crossing the highly porous borders of the three implicated countries, requiring intergovernmental coordination, with all the inherent logistical challenges in remote areas with poor infrastructure and communication networks and, in this case, significant language barriers.

**Figure 3:**
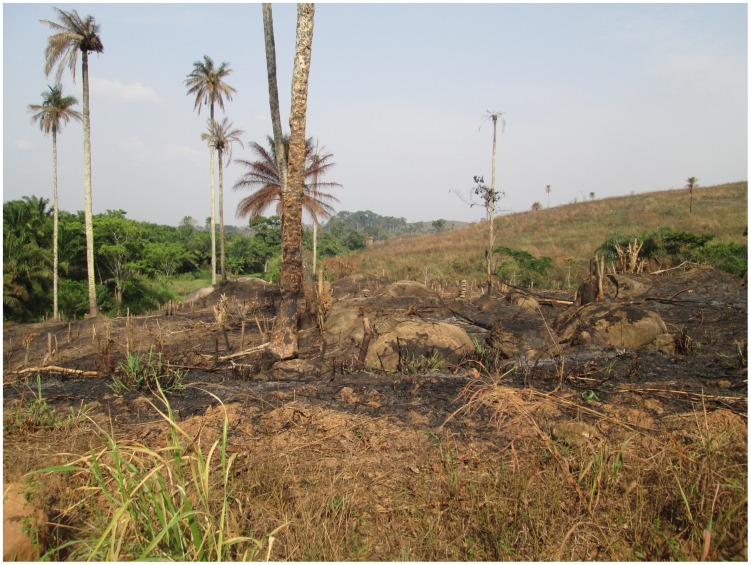
The area known as the Guinea Forest Region, now largely deforested because of logging and clearing and burning of the land for agriculture. Photo credit: Daniel Bausch.

Guinea, Liberia, and Sierra Leone, sadly, fit the bill for susceptibility to more severe outbreaks. While the devastating effects of the civil wars in Liberia and Sierra Leone are evident and well documented, readers may be less familiar with the history of Guinea, where decades of inefficient and corrupt government have left the country in a state of stalled or even retrograde development. Guinea is one of the poorest countries in the world, ranking 178 out of 187 countries on the United Nations Development Programme Human Development Index (just behind Liberia [174] and Sierra Leone [177]). More than half of Guineans live below the national poverty line and about 20% live in extreme poverty. The Guinea forest region, traditionally comprised of small and isolated populations of diverse ethnic groups who hold little power and pose little threat to the larger groups closer to the capital, has been habitually neglected, receiving little attention or capital investment. Rather, the region was systematically plundered and the forest decimated by clear-cut logging, leaving the “Guinea Forest Region” largely deforested (Figure 3).

**Figure 4:**
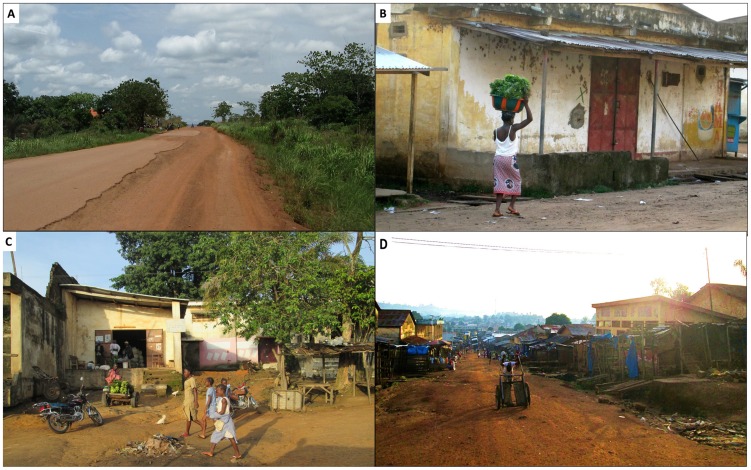
Scenes of the degraded infrastructure of the Guinea forest region. **A.** Once-paved, but now deteriorated road; **B, C, and D.** Street views of the dilapidated town of Guéckédou, the epicenter of the Ebola virus disease outbreak. Photos credit: Frederique Jacquerioz.

The forest region also shares borders with Sierra Leone, Liberia, and Cote d'Ivoire, three countries suffering civil war in recent decades. Consequently, the region has found itself home to tens of thousands of refugees fleeing these conflicts, adding to both the ecologic and economic burden. A United Nations High Commission for Refugees census of camps in the forest region in 2004 registered 59,000 refugees. Although the formal refugee camps have now been dismantled with improved political stability in the surrounding countries, the impact on the region is long lasting. Having worked in Guinea for a decade (1998–2008) on research projects based very close to the epicenter of the current Ebola virus outbreak, one of the authors (DGB) witnessed this “de-development” first-hand; on every trip back to Guinea, on every long drive from Conakry to the forest region, the infrastructure seemed to be further deteriorated—the once-paved road was worse, the public services less, the prices higher, the forest thinner (Figures 3 and 4).

Guinea fell further into governmental and civil disarray after former president Lansana Conté's death in 2008 left a power vacuum, with a series of coup d'états and periods of violence. Although the political situation has now somewhat stabilized, the country struggles to progress; socioeconomic indicators such as life expectancy (56 years) and growth national income (GNI) per capita ($440) have crept up in the past few years, but still remain disparagingly low. Despite a wealth of mineral and other natural resources, Guinea still possesses the eighth lowest GNI per capita in the world, and the incidence of poverty has been steadily increasing since 2003.

Lastly, why is this outbreak of Ebola virus happening now? As best as can be determined, the first case of Ebola virus disease in Guinea occurred in December 2013, at the beginning of the dry season, a finding consistent with observations from other countries that outbreaks often begin during the transition from the rainy to dry seasons [14]–[18]. Sharply drier conditions at the end of the rainy seasons have been cited as one triggering event [17]. Although more in-depth analysis of the environmental conditions in Guinea over the period in question remain to be conducted, inhabitants in the region do indeed anecdotally report an exceptionally arid and prolonged dry season, perhaps linked to the extreme deforestation of the area over recent decades. At present, we can only speculate that these drier ecologic conditions somehow influence the number or proportion of Ebola virus–infected bats and/or the frequency of human contact with them.

The precise factors that result in an Ebola virus outbreak remain unknown, but a broad examination of the complex and interwoven ecology and socioeconomics may help us better understand what has already happened and be on the lookout for what might happen next, including determining regions and populations at risk. Although the focus is often on the rapidity and efficacy of the short-term international response, attention to these admittedly challenging underlying factors will be required for long-term prevention and control.
